# *TP53* Mutational Analysis Enhances the Prognostic Accuracy of IHC4 and PAM50 Assays

**DOI:** 10.1038/srep17879

**Published:** 2015-12-16

**Authors:** Ching-Hung Lin, I-Chiun Chen, Chiun-Sheng Huang, Fu-Chang Hu, Wen-Hung Kuo, Kuan-Ting Kuo, Chung-Chieh Wang, Pei-Fang Wu, Dwan-Ying Chang, Ming-Yang Wang, Chin-Hao Chang, Wei-Wu Chen, Yen-Shen Lu, Ann-Lii Cheng

**Affiliations:** 1 Department of Oncology, National Taiwan University Hospital, Taipei, Taiwan; 2 Department of Internal Medicine; National Taiwan University Hospital, Taipei, Taiwan; 3 Oncology Center, National Taiwan University Hospital Hsin-Chu Branch, Hsin-Chu, Taiwan; 4 Department of Surgery, National Taiwan University Hospital, Taipei, Taiwan; 5 Graduate Institute of Clinical Medicine and School of Nursing, College of Medicine, National Taiwan University, Taipei, Taiwan; 6 International-Harvard Statistical Consulting Company, Taipei, Taiwan; 7 Department of Pathology, National Taiwan University Hospital, Taipei, Taiwan; 8 Department of Medical Research, National Taiwan University Hospital, Taipei, Taiwan; 9 Graduate Institute of Oncology and Cancer Research Centre, College of Medicine, National Taiwan University, Taipei, Taiwan

## Abstract

IHC4 and PAM50 assays have been shown to provide additional prognostic information for patients with early breast cancer. We evaluated whether incorporating TP53 mutation analysis can further enhance their prognostic accuracy. We examined TP53 mutation and the IHC4 score in tumors of 605 patients diagnosed with stage I–III breast cancer at National Taiwan University Hospital (the NTUH cohort). We obtained information regarding TP53 mutation and PAM50 subtypes in 699 tumors from the Molecular Taxonomy of Breast Cancer International Consortium (METABRIC) cohort. We found that TP53 mutation was significantly associated with high-risk IHC4 group and with luminal B, HER2-enriched, and basal-like subtypes. Despite the strong associations, TP53 mutation independently predicted shorter relapse-free survival (hazard ratio [HR] = 1.63, P = 0.007) in the NTUH cohort and shorter breast cancer-specific survival (HR = 2.35, P = <0.001) in the METABRIC cohort. TP53 mutational analysis added significant prognostic information in addition to the IHC4 score (∆ LR-χ^2^ = 8.61, P = 0.002) in the NTUH cohort and the PAM50 subtypes (∆ LR-χ^2^ = 18.9, P = <0.001) in the METABRIC cohort. We conclude that incorporating TP53 mutation analysis can enhance the prognostic accuracy of the IHC4 and PAM50 assays.

Breast cancer is one of the leading malignancies in women, with an increasing incidence over the past 2 decades. With the advent of screening, a large number of patients are being diagnosed at an early stage and have a favorable prognosis. However, these patients have a certain recurrence rate, depending on the clinicopathological features. Adjuvant chemotherapy can reduce the recurrence risk, but it has moderate adverse effects. The established clinicopathological features are insufficient to guide patients for adjuvant chemotherapy, and therefore substantial over- or under-treatment can occur.

Several multi-gene tests, such as MammaPrint^1^,^2^, Oncotype DX^3^,^4^, and PAM50^5^,^6^, have been shown to provide additional prognostic information over classical clinicopathological factors. To improve the applicability, Cuzick *et al.* constructed the IHC4 score by combining 4 widely examined immunohistochemical markers, namely, estrogen receptor (ER), progesterone receptor (PgR), human epidermal growth factor receptor 2 (HER2; including fluorescent *in situ* hybridization in the IHC 2+ group), and Ki-67^7^. Previous studies have shown that the IHC4 score provides similar prognostic information compared with the Oncotype DX recurrence score (RS)^7^ and PAM50 risk of recurrence score (ROR)^8^, and one study suggested that IHC4 has the potential to be the most cost-effective prognosis tool^9^.

Spearman correlations of risk groups defined by the RS, ROR, or IHC4 score are only modest to moderate (RS and IHC4 scores: *r* = 0.72; ROR and IHC4 scores: *r* = 0.48; RS and ROS scores: *r* = 0.39)^7^,^8^. Although a considerable difference exists among these 3 assays, combinations of 2 assays out of the 3 assays did not significantly improve the prognostic value^7^,^8^. A similar prognostic power, modest-to-moderate concordance, and the absence of a significant additive effect of these assays suggest the need for incorporating other factors to improve the prognostic accuracy.

The *PIK3CA* and *TP53* somatic mutations are the 2 most frequently mutated genes in breast cancer, and their frequencies are much higher than other somatic mutations^10^,^11^. In contrast to the conflicting findings about prognostic value of *PIK3CA* mutation^12^,^13^,^14^,^15^, *TP53* mutation has been consistently shown to predict poor outcomes in 2 meta-analyses (hazard ratio [HR] = 2.0 and 2.27)^16^,^17^. Mutant P53 in breast cancer may act at various cancer stages, such as early tumorigenesis, tumor growth, and metastasis. *TP53* mutation in breast cancer is associated with high-grade tumor behavior, and is molecularly distinct from wild type tumor^18^,^19^. MammaPrint, Oncotype DX, PAM50, and IHC4 mainly focus on measuring tumor proliferation on the basis of protein or mRNA expressions. The broad-spectrum effects of *TP53* mutation may be more likely to show additive effect on these assays. We determined whether incorporating *TP53* mutation can enhance the prognostic accuracy of the IHC4 and PAM50 assays.

## Methods

### Patients and sample collection

We evaluated the prognostic effect of *TP53* mutation in one retrospective cohort by using IHC4 scores and in one public data set by using PAM50 scores. The retrospective cohort included 659 patients with stage I–III breast cancer diagnosed at National Taiwan University Hospital (NTUH) from January 1997 to December 2005. Among them, 605 patients with adequate tumor DNAs for the *TP53* mutation analysis were enrolled in this study. This study was approved by the Ethics Committee of NTUH (201112010RIC). The informed consent was obtained from all subjects, and the methods used in this study were carried out in accordance with the approved guidelines. In this cohort, the clinicopathological data were extracted from medical charts. The relapse-free survival (RFS) data used in this study were current as of December 31, 2011. The RFS was defined as the duration from diagnosis to the confirmation of disease recurrence, including local, regional, and distant recurrences.

The Molecular Taxonomy of Breast Cancer International Consortium (METABRIC) dataset comprised 1992 patients with breast cancer from the United Kingdom and Canada, and data regarding clinicopathological features and PAM50 classification were publicly available for all patients^20^. From this dataset, we analyzed 699 patients with stage I–III breast cancer and publicly available *TP53* mutation status (METABRIC cohort).

### Classification of patients into risk groups according to the IHC4 scores in the NTUH cohort

In the NTUH cohort, tumors were stained for ER, PgR, and HER2 by using IHC as previously described^21^. The ER and PgR statuses were determined using the Ventana Benchmark system (Ventana Medical Systems Inc., Tucson, AZ, USA) and prediluted antibodies (anti-ER clone 6F11 and anti-PgR clone 16). ER and PgR were scored as percentage of tumor cells positively staining nuclei, and tumors with ≥10% positively stained cells were considered positive. The HER2 status was determined according to the American Society of Clinical Oncology/College of American Pathologists updated guideline^22^. Briefly, scores of 0 and 1+ by IHC were considered negative, and 3 + was considered positive. Cases with a score of 2+ were tested for gene amplification by dual probe fluorescence *in situ* hybridization. *HER2*/CEP17 ratio ≥2.0 and/ or an average *HER2* copy number ≥6.0 signals/cell were considered positive. The primary antibody for staining Ki67 was anti-Ki67 (1:200 dilution, clone MIB-1, DakoCytomation, Denmark)^23^,^24^, and tumors with ≥13.25% positively stained nuclei were considered as highly expressed^25^.

According to the study by Cuzick *et al.*^7^, the IHC4 score of each tumor was computed as IHC4 = 94.7 × (− 0.100 ∙ ER_10_ − 0.079 ∙ PgR_10_ + 0.586 ∙ HER2 + 0.240 ln [1 + 10 ∙ Ki67]). To avoid the bias caused by the differences in methodology and the antibodies between the present study and the study by Cuzick *et al.*^7^, we categorized our study participants into low, intermediate, and high risk groups according to the IHC4 scores of <25th, 25th–75th, and >75th percentiles, respectively.

### *TP53* mutational analysis in the NTUH cohort

In the NTUH cohort, *TP53* exons 4–9 were sequenced for each tumor, as previously described^26^. The hematoxylin and eosin stained slides of the tumors were examined, and the tumor areas were marked for macrodissection to enrich tumor DNAs. The genomic DNA of the macrodissected tumor specimens was isolated using the QIAamp DNA Mini Kit (Qiagen Inc., Valencia, CA, USA) and amplified using PCR. Forward and reverse sequencing of the amplified DNA was performed for the *TP53* exons 4–9 in an autosequencer (Applied Biosystems, USA) using sequencing or corresponding PCR primers.

### Clinicopathological data, PAM50 classification, and *TP53* mutational status in the METABRIC cohort

We extracted the data on demographics, survival, PAM50 classification, and the *TP53* mutation status from the METABRIC cohort. These data are listed in the supplementary Table 2 in the study by Curtis *et al.*^20^. Among the 1992 tumors, 820 tumors had *TP53* mutation. Because the METABRIC cohort did not have information on cancer relapse, we used breast cancer-specific survival (BCSS) as the endpoint. After excluding 9 patients with stage IV breast cancer, 17 patients without survival information, and 95 patients who died of conditions other than breast cancer, 699 patients were finally included in the analysis. In the METABRIC cohort, *TP53* mutations in all of the 11 exons were sequenced in forward and reverse directions as previously described^27^ (with the exception that exon 7 was sequenced in only one direction). According to the study by Parker *et al.*, the risk of relapse (ROR) score of each tumor was computed as per the following formula: 0.05 ∙ basal + 0.12 ∙ HER2 enriched − 0.34 ∙ luminal A + 0.23 ∙ luminal B ^5^.

### Statistical analysis

The distributional properties of categorical variables were presented as the frequency and percentage. The differences in the distributions of categorical variables between the *TP53* wild and mutant tumors of patients with breast cancer were examined using the chi-square test. The survival outcomes were estimated using the Kaplan–Meier method. In the univariate analysis, the effects of each potential predictive factor for the RFS outcome in the NTUH cohort and BCSS in the METABRIC cohort were examined using the log-rank test. Next, multivariate analysis was conducted by fitting Cox proportional hazards models to estimate the adjusted effects of predictors on the RFS and BCSS outcomes. Specifically, the stepwise variable selection procedure (with iterations between the forward and backward steps) was applied to obtain the most appropriate candidate for the final Cox proportional hazards model. To ensure quality, basic model-fitting techniques for (1) variable selection, (2) goodness-of-fit (GOF) assessment, and (3) regression diagnostics and remedies were performed in our regression analyses. To assess the prognostic effect of adding *TP53* mutation as a variable, we used changes in the likelihood ratio (LR) values (∆ LR-*χ*^2^) to quantitatively measure the relative score with *TP53* mutation information compared with that without *TP53* mutation information in the Cox model. A *P* value ≤ .05 was used to indicate statistical significance, and all tests were 2-tailed. Complete details of the statistical analysis are provided in the supplementary materials.

## Results

### Clinical and pathological characteristics of patients

**Figure 1:**
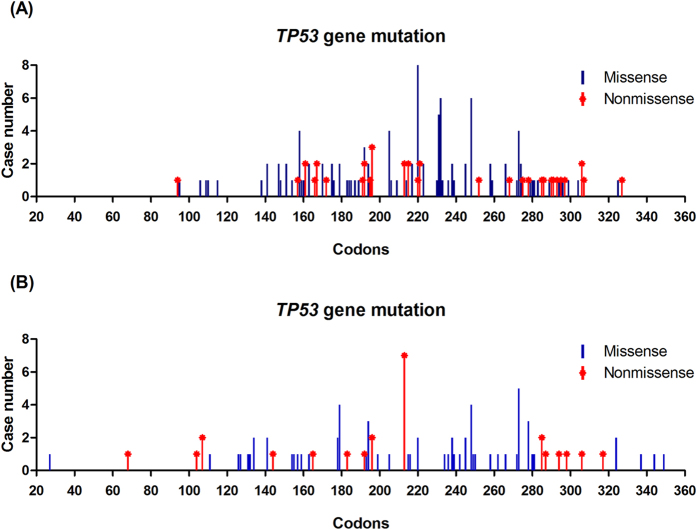
Distribution of missense and nonmissense mutations along the coding region of *TP53* in the NTUH cohort (**A**) and METABRIC (**B**) cohort.

The mutation distribution in the *TP53* coding regions in both cohorts are presented in Fig. 1. In the NTUH cohort, 153 mutations including 116 missense and 37 nonmisssense mutations were identified in 132 tumors. Some recurrently mutated codons, such as codons 220 (5.3%), 232 (3.9%), 248 (3.9%), and 231 (3.3%) were observed. In the METABRIC cohort, 90 mutations including 64 missense and 26 nonmisssense mutations, were identified in 90 tumors. Some recurrently mutated codons, such as codons 213 (7.6%), 273 (5.4%), 248 (4.3%), and 179 (4.3%) were observed. The clinical and pathological data of patients based on the *TP53* mutation status in the NUTH and the METABRIC cohorts are listed in Table 1. *TP53* mutation was associated with a high histological grade, ER negativity, and HER2 overexpression in both cohorts. In addition, *TP53* mutation was associated with PR negativity and a high Ki67 expression in the NUTH cohort and a high frequency of chemotherapy use in the METABRIC cohort. *TP53* mutation was not significantly associated with age, tumor size, or the axillary lymph node status of patients.

In the NTUH cohort, low, intermediate, and high risk groups were defined according to IHC4 scores of <−16.0, −16.0 to 119.7, and >119.7, respectively (Fig. S1). *TP53* mutation was significantly associated with higher risk groups on the basis of the IHC4 score (high vs. intermediate vs. low risk, 38% vs. 19% vs. 11%, *P* < 0.001). In the METABRIC cohort, the *TP53* mutation was significantly more common in HER2-enriched (25%) and basal-like (34%) subtypes than in luminal A (5%) and luminal B (13%) subtypes. Compared with luminal A subtype, luminal B subtype had significantly higher *TP53* mutation frequency. *TP53* mutation in the normal breast subtype was low (4%), but this mutation was observed in a relatively limited number of cases (Table IB). Furthermore, *TP53* mutation was significantly associated with a high ROR score (mean rank, *TP53* mutant vs. wild, 383.2 vs. 317.1, *P* = 0.001, according to a Mann–Whitney U test).

### Univariate survival analyses of prognostic factors

In the NTUH cohort, the median follow-up duration was 77.4 months (95% confidence interval [CI], 75.1–79.7 mo), and breast cancer relapse was observed in 144 patients. In the METABRIC cohort, the median follow-up duration was 120.5 months (95% CI, 111.9–129.1 mo) and 196 patients died of breast cancer. In the univariate analysis, the conventional clinicopathological factors including higher histological grade, higher T stage, higher N stage, and ER negativity were significantly associated with poor outcomes in the NTUH and the METABRIC cohorts. HER2 overexpression was marginally significantly and significantly associated with poor outcomes in the NTUH and the METABRIC cohorts, respectively. PR negativity and high Ki67 staining, which were unavailable in the METABRIC cohort, were significantly associated with shorter RFS in the NTUH cohort (Table S1).

**Figure 2:**
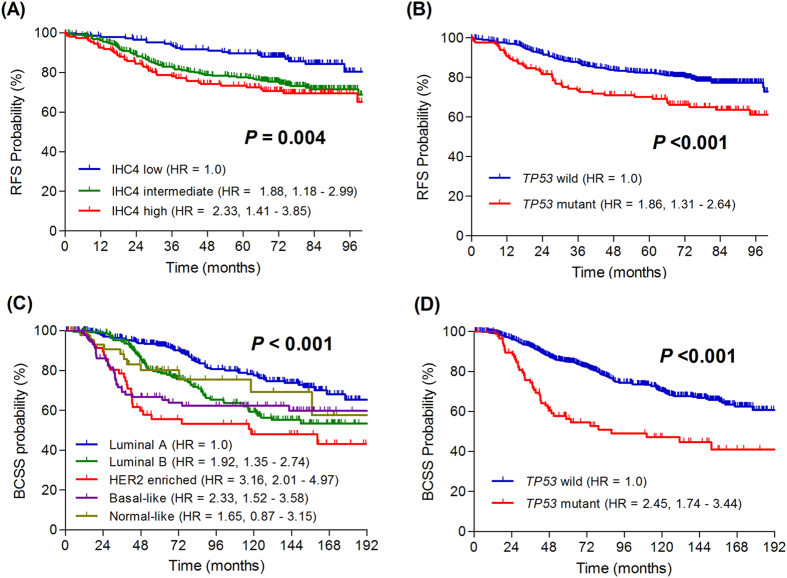
Kaplan-Meier plots of relapse-free survival by IHC4 risk classification (**A**) and *TP53* mutational status (**B**) in NTUH cohort, and breast cancer-specific survival by PAM50 classification (**C**) and *TP53* mutational status (**D**) in METABRIC cohort. (unadjusted analysis).

In the NTUH cohort, IHC4 intermediate (HR = 1.88, 95% CI = 1.18–2.99) and high risk (HR = 2.33, 95% CI = 1.41–3.85) groups were significantly associated with shorter RFS compared with the low risk group. In the METABRIC cohort, luminal B (HR = 1.92, 95% CI = 1.35–2.74), HER2-enriched (HR = 3.16, 95% CI = 2.01–4.97), and basal-like (HR = 2.33, 95% CI = 1.52–3.58) subtypes were associated with shorter BCSS compared with the luminal A subtype. The survival of normal breast subtype was not significantly different from that of the luminal A subtype. *TP53* mutation was significantly associated with shorter RFS (HR = 1.86, 95% CI = 1.31–2.64) in the NTUH cohort and with shorter BCSS (HR = 2.45, 95% CI = 1.74–3.44) in the METABRIC cohort (Fig. 2 and Table S1).

### Multivariate survival analyses of prognostic factors

In the NTUH cohort, we conducted the multivariate analyses separately by including each IHC4 marker or IHC4 risk group as a variable. Higher T and N stages were associated with shorter RFS in both analyses. When the IHC4 risk groups were used as a variable, both of the IHC4 high risk group (HR = 1.90, 95% CI = 1.32–2.73) and *TP53* mutation (HR = 1.63, 95% CI = 1.14–2.32) independently predicted shorter RFS (Table 2A). When each IHC4 marker was used as a variable and its cutoff was determined using a stepwise variable selection procedure, PR staining <10% (HR = 1.58, 95% CI = 1.11–2.24), Ki67 staining ≥10% (HR = 1.91, 95% CI = 1.33–2.76), and *TP53* mutation (HR = 1.53, 95% CI = 1.07–2.19) independently predicted shorter RFS (Table S2A).

In the METABRIC cohort, we conducted the multivariate analyses separately by including each PAM50 subtype or a continuous variable of ROR score as a variable. In both analyses, age <45 or >65 years, higher T stage, higher N stage, and HER2 overexpression were associated with shorter BCSS. When PAM50 subtypes were used as a variable, HER2-enriched (HR = 1.73, 95% CI = 1.04–2.87) and basal-like (HR = 1.74, 95% CI = 1.12–2.70) subtypes were significantly associated with shorter BCSS, and luminal B and normal breast subtypes were marginally associated with shorter BCSS compared with the luminal A subtype. *TP53* mutation independently predicted shorter BCSS (HR = 2.35, 95% CI = 1.64–3.36) (Table 2B). When ROR score (the continuous variable) was used as a variable, the multivariate analysis showed that an increased ROR score was significantly associated with shorter BCSS (HR = 2.03, 95% CI = 1.09–3.77). *TP53* mutation remained an independent predictive factor of shorter BCSS (HR = 2.46, 95% CI = 1.72–3.51) (Table S2B).

**Figure 3:**
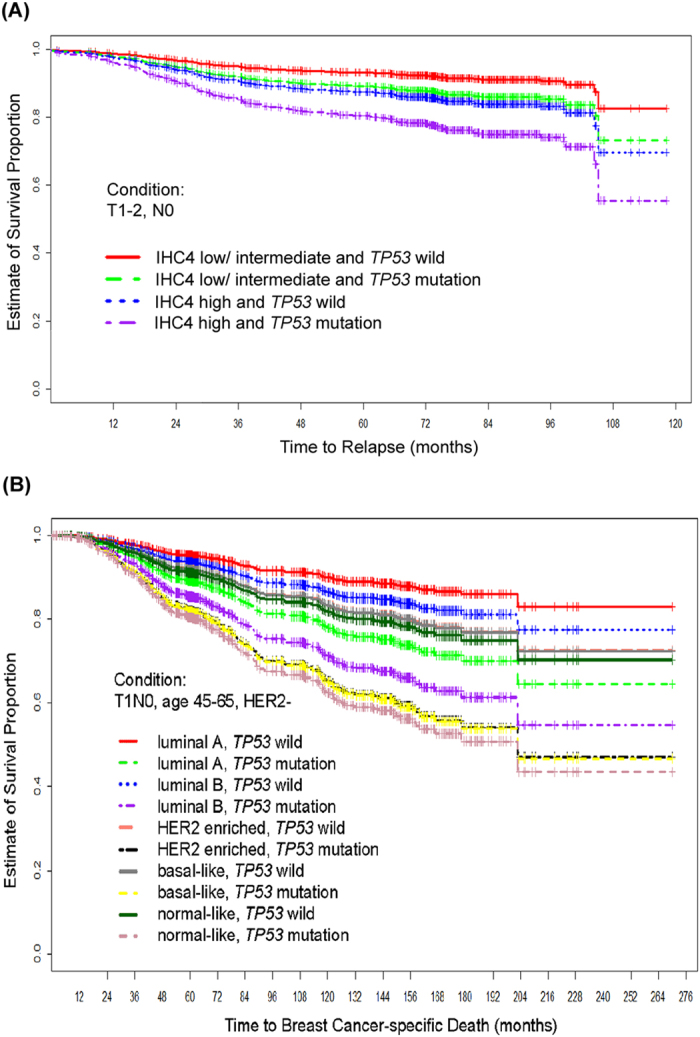
Covariate-adjusted survival curves for time to relapse in the NTUH (**A**) and METABRIC (**B**) cohorts.

*TP53* mutational analysis added significant prognostic information in addition to the IHC4 score (∆LR-χ^2^ = 8.61, *P* = 0.002) in the NTUH cohort, and PAM50 subtypes (∆ LR-χ^2^ = 18.9, *P* ≤ 0.001) in the METABRIC cohort (Table 2C). In view of the importance of lymph node status, we conducted subgroup analyses to assess whether the predicting effects of *TP53* mutation were different in lymph node negative and positive subgroups by adding two-way interactions of N stage (N0 vs. N1/ N2/ N3) and *TP53* mutation (mutant vs. wild) to the variable lists of the stepwise variable selection procedures in regression analyses in the two cohorts respectively. The interactive effects were not statistically significant in both Cox’s proportional hazards models reported in Tables 2A and 2B. It indicates that the predictive value of *TP53* mutation was not affected by lymph node status. To demonstrate the potential clinical utility, we drew covariate-adjusted survival curves that could facilitate fine tuning according to the association between the *TP53* mutational status and the IHC4 risk groups (Fig. 3A) or the PAM50 subtypes (Fig. 3B).

## Discussion

This study shows that *TP53* mutation is an independent prognostic factor beyond the IHC4 and PAM50 assays. Incorporating *TP53* mutation into these 2 assays can significantly enhance their prognostic accuracy.

In the present study, *TP53* mutation was significantly associated with IHC4 risk groups and PAM50 subtypes, but the Spearman correlations between *TP53* mutation and IHC4 or PAM50 (*TP53* mutation and IHC4: *r* = 0.24; *TP53* mutation and PAM50: *r* = 0.21) were lower than those between IHC4 and RS, ROR, and IHC4 in previous reports (RS and IHC4: *r* = 0.72; ROR and IHC4: *r* = 0.48; RS and ROS: *r* = 0.39)^7^,^8^. The relatively low Spearman correlations of *TP53* mutation with IHC4 and PAM50 and the independent prognostic value of *TP53* mutation strongly suggest that *TP53* mutation has a biology distinct from that of ER, PR, HER2, and markers of cell proliferation or PAM50 subtypes. Several previous studies have shown that in addition to the increase in cancer cell proliferation, mutant P53 can cause other effects, such as genomic instability, inflammation, angiogenesis, epithelial-to-mesenchymal transition, and stemness^18^,^28^,^29^,^30^,^31^,^32^,^33^.

Although the IHC4 score is widely applicable and its prognostic accuracy is similar to that of RS^7^ and ROR^8^ scores, the major drawback of using the IHC4 score is the lack of standardization of the Ki67 assay and its interpretation. To evaluate Ki67 expression, we used the well-known MIB-1 antibody^34^. A prior study showed a strong correlation between MIB-1 and SP6 antibodies, and no adjustment was required^35^. However, Cuzick *et al.* used the SP6 antibody to generate an IHC4 score in an exploratory cohort (ATAC trial) and the MIB-1 antibody in a validation cohort (Nottingham data) and reported that the adjustment should be made using a factor of approximately 2.5 when the MIB-1 antibody is used^7^. Another limitation of the Ki67 assay is the lack of consistency across laboratories; for example, an international Ki67 reproducibility study reported high intralaboratory reproducibility but only moderate interlaboratory reproducibility^36^. To reduce these biases, we conducted multivariate analyses separately by including the IHC4 risk group or each IHC4 marker as a variable. Both of the multivariate analyses consistently showed that *TP53* mutation was an independent prognostic factor (HR = 1.63 when the IHC4 risk group was considered a variable and HR = 1.53 when each IHC4 marker was considered a variable) (Table 2A and Table S2A).

Among the 5 PAM50 subtypes, the normal-like subtype represented the gene signature close to true “normals”, resulting from reduction mammoplasty or grossly uninvolved tissue^5^. Because the normal-like subtype was considered a poor quality-control measure, it was excluded from the ROR score. Conservatively, we conducted the multivariate analyses separately by including each subtype or the ROR score as a variable in the METABRIC cohort. Both of the multivariate analyses consistently showed that *TP53* mutation was an independent prognostic factor (HR = 2.35 when each subtype was considered a variable and HR = 2.46 when the ROR score was considered a continuous variable, Table 2B and Table S2B). Recently, Silwal-Pandit *et al.* obtained data on 1460 tumors from a METABRIC cohort and sequenced all of the *TP53* exons. Their findings were consistent with those of our study, in which *TP53* mutation caused significantly inferior BCSS (HR = 2.03). Conversely, the PAM50 subtype was not included as a variable in the multivariate analysis in their study^37^.

Different types of *TP53* mutations have been reported as having different functional effects and prognostic values^38^,^39^. However, reports of these associations are inconsistent^17^,^37^,^40^,^41^,^42^,^43^. In our study, missense and nonmissense mutations were significantly associated with RFS (missense mutation vs. wild; HR = 1.80; nonmissense mutation vs. wild, HR = 2.27) in the NTUH cohort (Fig. S2A) and with BCSS (missense mutation vs. wild, HR = 2.42; nonmissense mutation vs. wild, HR = 2.51) in the METABRIC cohort (Fig. S2B). We examined the prognostic value of recurrently mutated codons 220, 232, 248, and 231 in the NTUH cohort and 213, 273, 248, and 231 in the METABRIC cohort. None of these codons reached the statistical significance to predict patient outcomes when compared with the mutations of a pool of other codons (data not shown). Because the case number was limited by each codon, we could not exclude the possibility of different prognostic effects exerted by specific mutated codons.

In conclusion, the novel finding of the present study is that *TP53* mutation has additional predictive value that isn’t captured in proliferation-oriented IHC4 and PAM50 platforms. To confirm the findings of this study is crucial for supporting the routine clinical application of *TP53* mutation as outcome predictor for patients with early breast cancer. Furthermore, the findings warrant conducting studies to develop new platforms by incorporating *TP53* mutation to IHC4 and PAM50 respectively, and to test the prognostic value of *TP53* mutation when other assays, such as Oncotype DX and MammaPrint, are used.

## Additional Information

**How to cite this article**: Lin, C.-H. *et al.* *TP53* Mutational Analysis Enhances the Prognostic Accuracy of IHC4 and PAM50 Assays. *Sci. Rep.* **5**, 17879; doi: 10.1038/srep17879 (2015).

## Supplementary Material

Supplementary Information

## Figures and Tables

**Table 1:**
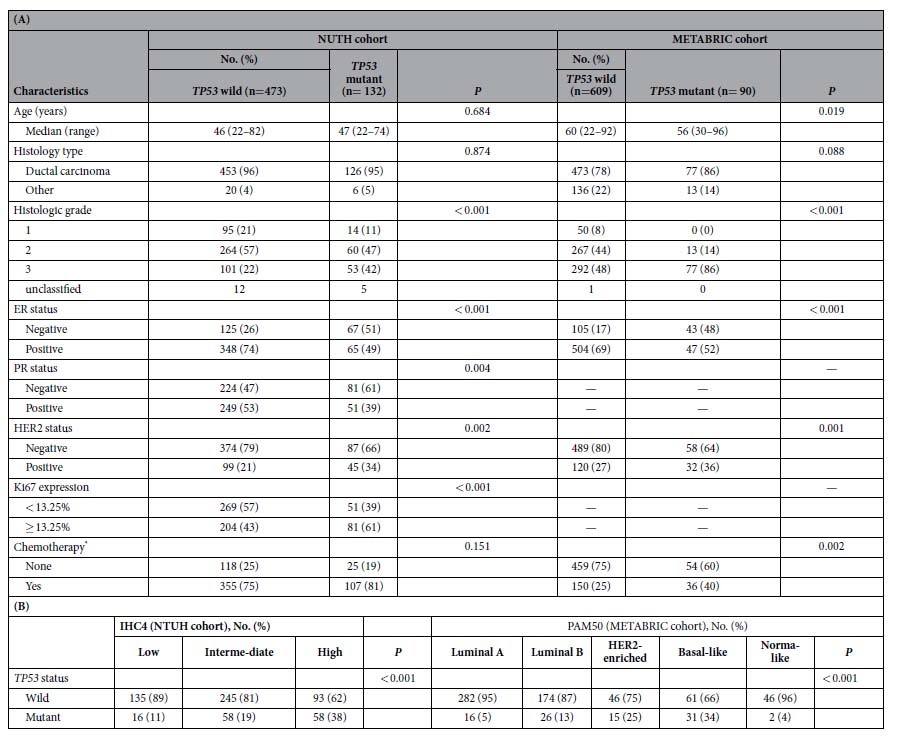
Correlations of *TP53* mutation status with clinicopathological characteristics (A) and IHC4 risk group and PAM50 subtypes (B).

ER, estrogen receptor; PR, progesterone receptor; HER2, human epidermal growth factor receptor 2. *Neoadjuvant and/or adjuvant therapy.

**Table 2:** Multivariate Cox’s proportional hazards models for relapse-free survival in NTUH cohort (A) and breast cancer-specific mortality in METABRIC cohort (B) and comparison of added prognostic information by *TP53* in the two cohorts (C).

(A) Relapse-free survival			
Characteristic	HR	95% CI	*P*
T stage			
T3 *v* T1 / T2	2.65	1.78–3.94	<0.001
N stage			
N0 *v* N1 / N2	0.47	0.32–0.70	<0.001
N3 *v* N1 / N2	1.64	1.05–2.57	0.030
IHC4 score			
High/intermediate *v* low	1.90	1.32–2.73	<0.001
*TP53* status			
Mutant *v* wild	1.63	1.14–2.32	0.007
**(B)** Breast cancer-specific mortality			
Age			
<45/ >65 *v* 45–65 years	1.71	1.27–2.29	<0.001
T stage (ordinal)			
Increased one unit (1 *v* 2 *v* 3)	1.45	1.11–1.91	0.007
N stage (ordinal)			
Increased one unit (0 *v* 1 *v* 2. *v* 3)	1.58	1.33–1.87	<0.001
HER2 overexpression			
Yes *v* no	1.58	1.12–2.23	0.010
PAM50			
Luminal B *v* luminal A	1.38	0.95–1.99	0.090
HER2 enriched *v* luminal A	1.73	1.04–2.87	0.036
Basal-like *v* luminal A	1.74	1.12–2.70	0.013
Normal-like *v* luminal A	1.90	0.99–3.65	0.054
*TP53* status			
Mutant *v* wild	2.35	1.64–3.36	<0.001
**(C)**			
**Cohort**	**∆ LR-χ^2^**	*P*	
NTUH			
T stage + N stage + IHC4 + TP53 *v* T stage + N stage + IHC4 (df=1)	1668.3 − 1659.7 = 8.6	0.002	
METABRIC			
age + T stage + N stage + HER2 + PAM50 + TP53 *v* age + T stage + N stage + HER2 + PAM50 (df=1)	2261.3 − 2242.4 = 18.9	< 0.001	
