# Supplementary material for: TP53 Mutational Analysis Enhances the Prognostic Accuracy of IHC4 and PAM50 Assays
Source: Sci Rep. 2015 Dec 16;5:17879. doi: 10.1038/srep17879 (PMC4680865; doi:10.1038/srep17879)

TP53 Mutational Analysis Enhances the Prognostic Accuracy of IHC4 and PAM50 Assays

Ching-Hung Lin, I-Chiun Chen, Chiun-Sheng Huang, Fu-Chang Hu, Wen-Hung Kuo, Kuan-Ting Kuo, Chung-Chieh Wang, Pei-Fang Wu, Dwan-Ying Chang, Ming-Yang Wang, Chin-Hao Chang, Wei-Wu Chen, Yen-Shen Lu, Ann-Lii Cheng

Supplementary Information

Supplementary materials

Detailed procedure of statistical analysis

Statistical analysis was performed using the R 3.1.1 software (R Foundation for Statistical Computing, Vienna, Austria). In statistical testing, two-sided p value ≤ 0.05 was considered statistically significant. The distributional properties of categorical variables were presented by frequency and percentage, and the survival curves of survival outcomes were estimated by the Kaplan-Meier method. The differences in the distributions of categorical variables between the *TP53* wild and mutant groups of patients with breast cancer were examined using chi-square test. In the univariate analysis, the effects of each potential predictive factor for the relapse-free survival (RFS) outcome in NTUH cohort and breast cancer specific survival (BCSS) were examined using log-rank test. Next, multivariate analysis was conducted by fitting Cox's proportional hazards models to estimate the adjusted effects of predictors on the RFS and BCSS outcome.

The goal of regression analysis was to find one or a few parsimonious regression models that fitted the observed data well for effect estimation and/or outcome

prediction. To ensure the analysis quality, basic model-fitting techniques for (1) variable selection, (2) goodness-of-fit (GOF) assessment, and (3) regression diagnostics and remedies were used in our regression analyses. Specifically, the *stepwise variable selection procedure* (with iterations between the *forward* and *backward* steps) was applied to obtain the best candidate final Cox's proportional hazards model. All the univariate significant and non-significant relevant covariates and some of their interactions were put on the variable list to be selected. The significance levels for entry (SLE) and for stay (SLS) were set to 0.15 (or larger) for being conservative. Then, with the aid of substantive knowledge, the best candidate final Cox's proportional hazards model was identified manually by dropping the covariates with p value > 0.05 one at a time until all regression coefficients were significantly different from 0. Any discrepancy between the results of univariate analysis and multivariate analysis was likely due to the confounding effects of the uncontrolled covariates in univariate analysis.

The GOF measures, *concordance* and *adjusted generalized* R^2, were examined to assess the GOF of the fitted Cox's proportional hazards model. The concordance of Cox's proportional hazards model is equivalent to the estimated area under the receiver operating characteristic (ROC) curve (i.e., the c statistic) for logistic regression model, and thus its value ≥ 0.7 suggests an acceptable level of discrimination power. Yet, the value of adjusted generalized R^2 ($0 \leq R^2 \leq 1$), proposed by Nagelkerke (1991), is usually low for Cox's proportional hazards model — in our experience, adjusted generalized $R^2 \geq 0.15$ indicates an acceptable fit for Cox's proportional hazards model.

Simple and multiple generalized additive models (GAMs) were fitted to detect nonlinear effects of continuous covariates and identify appropriate cut-off point(s) for

discretizing continuous covariates, if necessary, during the stepwise variable selection procedure. Computationally, the vgam function (with the default values of smoothing parameters) of the VGAM package (Yee and Wild, 1996; Yee, 2013) was used to fit GAMs for continuous, binary, and count responses in R. Since GAMs were originally developed for smoothing the effects of continuous covariates in generalized linear models (GLMs), we fitted GAMs of binary response (i.e., 1 = relapse vs. 0 = not relapse) for our RFS outcome. Finally, the statistical tools of regression diagnostics for verification of proportional hazards assumption, residual analysis, detection of influential cases, and check of multicollinearity were applied to discover any model or data problems. The values of variance inflating factor (VIF) $\geq$ 10 in continuous covariates or $\geq$ 2.5 in categorical covariates indicate the occurrence the multicollinearity problem among some of the covariates in the fitted Cox's proportional hazards model.

References

Nagelkerke, N. (1991). A note on a general definition of the coefficient of determination. *Biometrika*, 78(3), 691-692.

Yee, T. W. (2013). VGAM: Vector generalized linear and additive models. R package, version 0.9-2 (URL: http://CRAN.R-project.org/package=VGAM).

Yee, T. W. and Wild, C. J. (1996). Vector generalized additive models. *Journal of Royal Statistical Society*, Series B, 58(3), 481-493.

Table S1 Univariate analysis of correlation of clinicopathologic variables with relapse-free survival in NTUH cohort and breast cancer-specific survival in METABRIC cohort

	DFS in NTUH cohort			BCSS in METABRIC cohort		
	HR	95% CI	*P*	HR	95% CI	*P*
Age			.399			< .001
<45 years	1.00			1.00		
45-65 years	0.59	0.26-1.37		0.58	0.40-0.84	
>65	0.87	0.62-1.21		1.05	0.73-1.50	
Histology grade			.007			< .001
1	1.00			1.00		
2	1.62	0.96-2.75		1.28	0.61-2.68	
3	2.30	1.32-4.00		2.64	1.29-5.39	
Tumor size			< .001			< .001
≤ 2 cm	1.00			1.00		
2-5 cm	1.87	1.23-2.84		1.97	1.45-2.67	
> 5 cm	6.24	3.92-9.92		3.45	1.81-6.56	
Axillary lymph node			< .001			< .001
None or cN0	1.00			1.00		
1-3 or cN1	2.56	1.69-3.86		1.82	1.30-2.55	
4-9 or cN2	2.63	1.58-4.38		3.68	2.52-5.37	
≥ 10 or cN3	5.46	3.34-8.93		5.85	3.25-10.53	
ER status			.004			< .001
Negative	1.00			1.00		
Positive	0.61	0.44-0.85		0.51	0.38-0.69	
PR status			< .001			—
Negative	1.00			—	—	
Positive	0.50	0.35-0.70		—	—	
HER2 status			.064			< .001
No	1.00			1.00		
Yes	1.41	0.98-2.01		2.00	1.48-2.71	
Ki67 expression			< .001			—
<10%	1.00			—	—	
≥10%	1.87	1.34-2.61		—	—	
TP53 status			< .001			< .001
wild	1.00			1.00		
mutant	1.86	1.31-2.64		2.45	1.74–3.44	
IHC4 score			.004			—
Low	1.00			—	—	
Intermediate	1.88	1.18-2.99		—	—	

High	2.33	1.41-3.85		—	—	
PAM50 classification						< .001
Luminal A			—	1.00		
Luminal B	—	—		1.92	1.35-2.74	
HER2 enrich	—	—		3.16	2.01-4.97	
Basal-like	—	—		2.33	1.52-3.58	
Normal-like	—	—		1.65	0.87-3.15	

RFS, relapse-free survival; BCSS, breast cancer-specific survival; HR, hazard ratio; CI, confidence interval; ER, estrogen receptor; PR, progesterone receptor; HER2, human epidermal growth factor receptor 2.

Table S2 Multivariate Cox hazard regression models of relapse-free survival with each IHC marker as a variable in NTUH cohort (A) and breast cancer-specific mortality with risk of recurrence (ROR) score in METABRIC cohort (B)

(A)

	Relapse-free survival		
**Characteristic**	HR	95% CI	*P*
Tumor size			
T3 *v* T1/ T2	2.83	1.90-4.20	< .001
Lymph node			
N0 *v* N1/ N2	0.49	0.33-0.73	< .001
N3 *v* N1/ N2	1.83	1.17-2.87	.009
PR staining			
<10% *v* ≥10%	1.58	1.11-2.24	.011
Ki67 staining			
≥10% *v* <10%	1.91	1.33-2.76	< .001
IHC4 score			
High *v* low/ intermediate	—	—	—
TP53 status			
Mutant *v* wild	1.53	1.07-2.19	.019

(B)

	Breast cancer-specific mortality		
**Characteristic**	HR	95% CI	*P*
Age			
<45/ >65 *v* 45-65 years	1.61	1.19-2.17	.002
T stage (ordinal)			
Increased one unit (1 *v* 2 *v* 3)	1.54	1.17-2.04	.003
N stage (ordinal)			
Increased one unit (0 *v* 1 *v* 2. *v* 3)	1.56	1.31-1.85	< .001
HER2 overexpression			
Yes *v* no	1.45	1.04-2.01	.027
PAM50			
Luminal B *v* luminal A	—	—	—
HER2 enriched *v* luminal A	—	—	—
Basal-like *v* luminal A	—	—	—
Normal-like *v* luminal A	—	—	—
ROR score (continuous)			
Increased one unit	2.03	1.09-3.77	.025
TP53 status			
Mutant *v* wild	2.46	1.72-3.51	< .001

Figure S1 Histogram of IHC4 score with median and interquartile range in the NTUH cohort. Q, quartile.

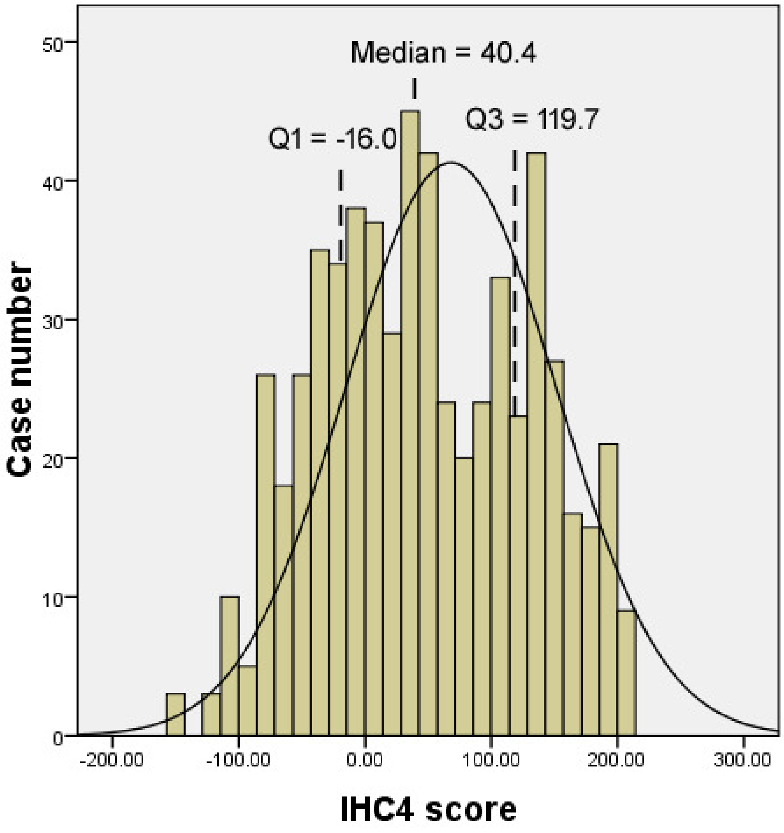

Figure S2 Kaplan-Meier plots of relapse-free survival by type of *TP53* mutation in NTUH cohort (A), and breast cancer-specific survival by type of *TP53* mutation in METABRIC cohort (B). (unadjusted analysis)

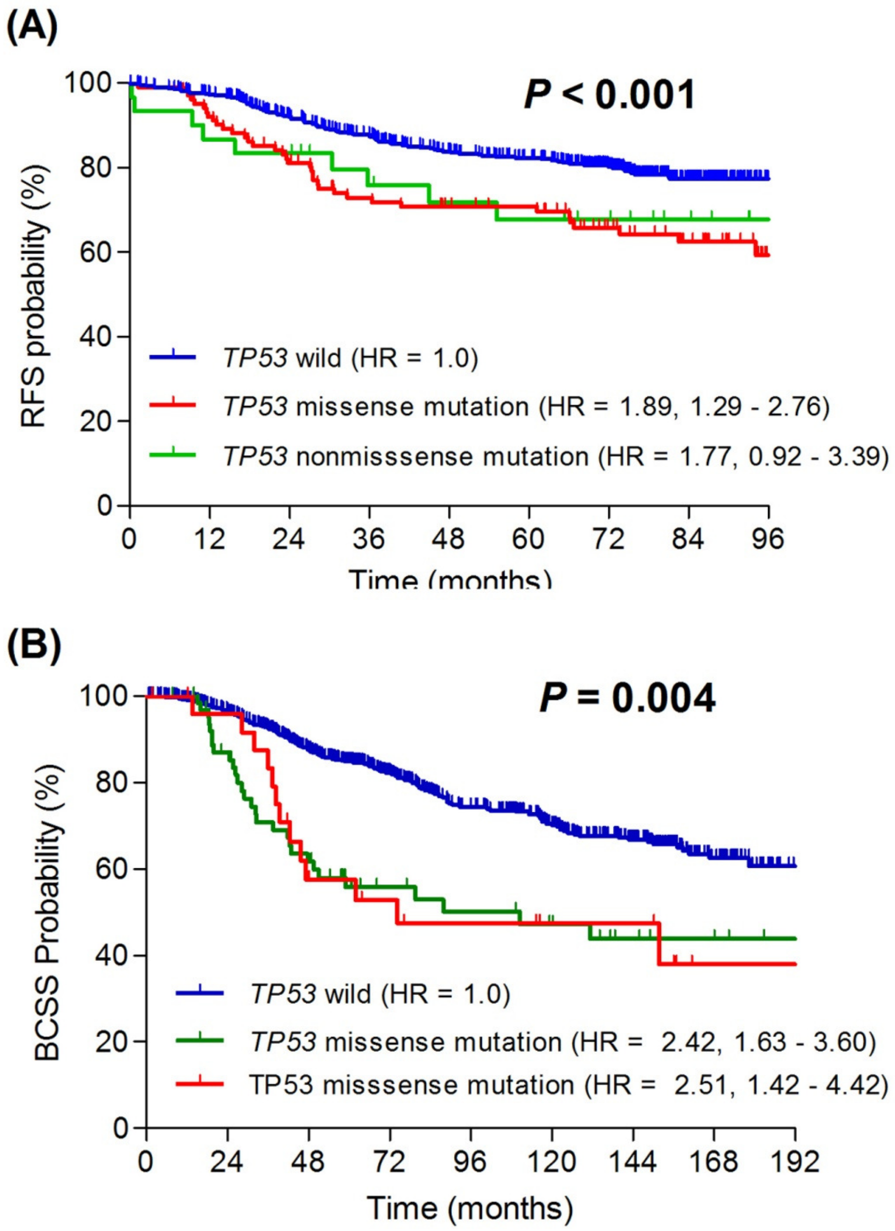

Supplement: Supplementary Information [file srep17879-s1.pdf]
